# Elimination of rheumatoid synovium *in situ *using a Fas ligand 'gene scalpel'

**DOI:** 10.1186/ar1811

**Published:** 2005-09-08

**Authors:** Haidi Zhang, Guangping Gao, Gilda Clayburne, H Ralph Schumacher

**Affiliations:** 1 Division of Pharmaceutics and Industry Pharmacy, School of Pharmacy, Long Island University, Brooklyn, NY, USA; 2 Department of Medicine, School of Medicine, University of Pennsylvania, Philadelphia, PA, USA; 3 Veterans Affairs Medical Center in Philadelphia, Philadelphia, PA, USA

## Abstract

Surgical synovectomy to remove the inflammatory synovium can temporarily ameliorate rheumatoid inflammation and delay the progress of joint destruction. An efficient medically induced programmed cell death (apoptosis) in the rheumatoid synovium might play a role similar to synovectomy but without surgical tissue damage. Gene transfer of Fas ligand (FasL) has increased the frequency of apoptotic cells in mouse and rabbit arthritic synovium. In this study, we investigated whether repeated FasL gene transfer could remove human inflammatory synovial tissue *in situ *and function as a molecular synovectomy. Briefly, specimens of human synovium from joint replacement surgeries and synovectomies of rheumatoid arthritis (RA) patients were grafted subcutaneously into male C.B-17 severe combined immunodeficiency (SCID) mice. Injections of a recombinant FasL adenovirus (Ad-FasL) into the grafted synovial tissue at the dosage of 10^11 ^particles per mouse were performed every two weeks. Three days after the fifth virus injection, the mice were euthanized by CO_2 _inhalation and the human synovial tissues were collected, weighed and further examined. Compared to the control adenovirus-LacZ (Ad-LacZ) and phosphate buffered saline (PBS) injected RA synovium, the Ad-FasL injected RA synovium was dramatically reduced in size and weight (P < 0.005). The number of both synoviocytes & mononuclear cells was significantly reduced. Interestingly, an approximate 15-fold increased frequency of apoptotic cells was observed in RA synovium three days after Ad-FasL injection, compared with control tissues. In summary, our *in vivo *investigation of gene transfer to human synovium in SCID mice suggests that repeated intra-articular gene transfer of an apoptosis inducer, such as FasL, may function as a 'gene scalpel' for molecular synovectomy to arrest inflammatory synovium at an early stage of RA.

## Introduction

Rheumatoid arthritis (RA) is a potentially very disabling disease that is characterized by chronic synovitis, a hyperplasic synovial membrane, and finally cartilage and bone destruction. Overgrowth of fibroblast-like synoviocytes as well as their secretion of an impressive array of cytokines/chemokines, adhesion molecules and proteases play important roles in the pathogenesis of rheumatoid joint destruction [1-3]. Surgical synovectomy to remove the inflammatory synovium can temporarily ameliorate rheumatoid inflammation and delay the progress of joint destruction [4]. An efficient medically induced programmed cell death (apoptosis) in the inflammatory synovium [5-7] might play a role similar to surgical synovectomy.

Hyperplasia of the rheumatoid synovium may result from the imbalance between cell proliferation and apoptosis. Mutations in tumor suppressor genes such as *p53*, and elevated expression of proto-oncogenes and apoptosis inhibitors, such as c-myc, c-fos, c-ras, c-jun, and bcl-2 in RA synoviocytes, may lead to inadequate apoptosis and tumor-like proliferation of rheumatoid synoviocytes [8-11]. Thus, the induction of apoptosis by gene transfer of an apoptosis inducer or growth modulator in a sense or anti-sense orientation may function as a 'gene scalpel' for decreasing or eliminating the hyperplasia of the synovial membrane.

Fas, a membrane-bound death molecule, is highly expressed in RA synovial lining and sub-lining cells compared to osteoarthritis and normal synovial tissues [11]. Between 30% and 90% of cells in RA synovium are positive for Fas antigen, but Fas ligand (FasL) mRNA has been detected only in mononuclear cells in RA synovium [10]. The upregulation of Fas-mediated apoptosis in inflammatory synovium has been established by intra-articular administration of adenovirus mediated FasL gene [5,12], anti-Fas monoclonal antibodies [13-15], and FasL transfectants [16]. Current reports indicate that a medically induced upregulation of the Fas-mediated apoptosis pathway in inflammatory synoviocytes may provide a novel therapeutic strategy for RA treatment [5,12-18].

Compared to other current available gene delivery vehicles, adenoviral vector has high transduction efficiency into human and rabbit fibroblast-like synoviocytes in vitro and into the synovium of mice, rabbits, guinea pigs and rhesus monkeys in vivo (5,12,19,7). Thus, recombinant adenovirus could be an ideal vector for identifying dose dependent effects of certain transgenes expressed in arthritic joints, although it would not be an ideal vehicle for molecular synovectomy in clinical administration because of its strong immunogenicity.

Induction of apoptosis in RA synoviocytes by gene transfer may be an efficient approach for the treatment of synovitis because the inflammatory cytokines/chemokines and proteases/adhesion molecules in RA synovium will be limited once the producing cells have died. Determination of the effects of FasL gene transfer on human inflammatory synovium *in vivo *is an important step in progressing from mouse/rabbit gene therapies to human gene therapy. A novel experimental system incorporating the grafting of RA synovial tissue into severe combined immunodeficiency (SCID) mice [20-22] has provided an *in vivo *model for the evaluation of our hypothesis; the repeated gene transfer of an apoptosis inducer such as FasL, mediated by an efficient vehicle, might function as a gene scalpel for the removal of inflammatory synovium *in situ*.

## Materials and methods

### Rheumatoid arthritis synovial tissues

Synovium and cartilage were obtained from RA patients who were diagnosed according to the 1987 revised criteria of the American College of Rheumatology [23] and who underwent joint replacement surgeries and synovectomies. The fresh RA synovium and cartilage used for grafting into SCID mice were obtained from the same RA patient at the time of surgery, in accordance with the Institutional Review Board approved study protocol at University of Pennsylvania. The human tissues were kept on ice during the procedures. RA synovium from the knee, hip, or shoulder joints of six patients were examined for the effects of FasL gene transfer *in vivo *in SCID mice.

### Animal model

Male C.B-17 SCID mice (Taconic, Germantown, NY, USA) aged 6 to 7 weeks were housed in the Research Animal Facility at the Veterans Affairs Medical Center in Philadelphia. SCID mice were kept at 72–75°F and handled under specific pathogen-free conditions. During surgical procedures, the mice were anesthetized intra-peritoneally with ketamine 2 mg and xylazine 0.4 mg in 0.2 ml PBS per mouse. RA synovial tissues were cut into 2 × 3 × 3 mm^3 ^pieces. Synovium alone or synovium mixed with cartilage were grafted into SCID mice at 200 mg tissue per mouse subcutaneously on their backs approximately 30 minutes after the synovial tissues were removed from patients. The entire procedure was performed under sterile conditions.

### Adenovirus preparation

The FasL adenovirus (Ad-FasL) and the LacZ adenovirus (Ad-LacZ) were generated as described previously [5,24] based on human type 5 adenovirus vector with the deletion of E1a, E1b, and a portion of the E3 region. The cDNA of FasL or LacZ was inserted between the cytomegalovirus enhancer/promoter and simian virus 40 late gene polyadenylation signal, respectively, in the 5' inverted terminal repeat regions of adenovirus type 5 at the place of the E1 deletion. Stock viruses were amplified in 293 cells with a variety of virus titer and serum concentrations. After 36 to 60 h of virus infection, cells were harvested and lysed by freeze and thaw cycles and purified through a two-round of cesium chloride gradient centrifugation. The cesium chloride was removed by a BioRad desalting column (Bio-Rad Laboratories, Hercules, CA, USA). The viruses were diluted with 10% glycerol in PBS (pH 7.4) to 2–4 × 10^12 ^particles per ml, aliquoted and frozen at -80°C until use. The transducibility of Ad-LacZ and Ad-FasL was evaluated by detection of β-galactosidase transgene expression and apoptosis induction in human synovial fibroblasts *in vitro*.

### Tissue X-gal staining

To detect the adenovirus vector mediated dose dependent transgene expression *in vivo*, RA synovium alone or with cartilage *in vivo *in the SCID mice were injected with Ad-LacZ at 10^9^, 10^10 ^or 10^11 ^particles per mouse into the middle of the grafted tissues. Three days after virus injection, the synovium alone or with cartilage was collected and fixed in 4% paraformaldehyde on ice for 2 h, then washed three times with PBS and stained in a solution of 5 mM K_4_Fe(CN)_6_, 5 mM K3 Fe(CN)_6_, 2 mM MgCl_2 _in PBS containing 0.5 mg/ml of X-gal stain (5-bromo-4-chloro-3-indolyl-b-D-galactopyranoside; Sigma Chemical Co., St Louis, MO, USA) overnight at 37°C. Samples were then washed three times with PBS, photographed and embedded in paraffin blocks.

### Treatment of the rheumatoid arthritis-SCID model

To identify the clinical potential of FasL gene transfer, RA synovium from six patients was grafted into SCID mice at 200 mg tissue per mouse subcutaneously. Two weeks after engraftment, 10^11 ^particles per mouse of Ad-FasL or Ad-LacZ in 0.1 ml PBS, or 0.1 ml PBS only, were injected into the engrafted tissue location in the SCID mice. The injections were repeated every two weeks for a total of five times. Mice were euthanized by CO_2 _inhalation three days after the fifth virus injection. The human synovial tissues were collected, weighed, and used for further examinations. Ten samples from each treatment group were examined. The weight of each sample was measured individually for each group as shown in Table 1. Comparative statistical analysis of the effects of Ad-LacZ and Ad-FasL treatments on the weight of RA synovium was performed using the rank sum test. Some grafted tissues were collected three days after the first virus treatment for the detection of apoptosis and Fas and FasL protein expression in synovial cells.

### Tissue hematoxylin and eosin staining

RA synovium and cartilage removed from the SCID mice were fixed in 10% phosphate-buffered formalin. After paraffin embedding, tissue sections (6 μm) were stained with hematoxylin and eosin for morphological evaluation.

### Reverse transcription-PCR

Total RNA was isolated from the grafted RA synovium using the Ultraspectrum RNA System (Biotech Laboratories, Houston, TX, USA). The specific first-strand cDNA synthesis and amplification were performed in the one tube for two-step reactions with the Promega Access Systems (Promega Corp., Madison, WI, USA). The specific primers used for detection of the transgenic FasL mRNA expression correspond to the coding region sequence of FasL cDNA (forward primer: 5'-TCAGCTCTTCCACCTGCAG-3') and poly(A) region sequence of the viral vector (reverse primer: 5'-CACTGCATTCTAGTTGTGG-3') to generate a 688 base-pair (bp) DNA fragment. The pair of primers corresponding to β-actin cDNA (forward primer: 5'-GAAATCGTGCGTGACATTAAG-3' ; reverse primer: 5'-CTAGAAGCATTTGCGGTGGACG-3') was used to generate a 505 bp cDNA product as an internal control to evaluate the quality of RNA samples. Briefly, the first-strand cDNA was synthesized for 45 minutes at 48°C, and then amplified through 35 cycles at 94°C for 1 minute, 55°C for 1 minute and 72°C for 2 minutes. The final concentration of reagents was 1.5 mM MgSO_4_, 0.2 mM each dNTP, 1 μM each primer, 0.1u/ μl AMV reverse transcriptase and 0.1u/ μl Tfl DNA polymerase obtained from Promega Corp. Subsequently, aliquots of the PCR products were fractionated by electrophoresis on a 1% agarose gel and visualized by ethidium bromide staining. The PCR products of transgenic FasL cDNA were then purified from the gel using the QIAquick Gel Extraction Kit (QIAGEN, Valencia, CA, USA) and were confirmed by sequencing analysis using BigDye Terminator Ready Reaction Kits (PE Biosystems, Foster City, CA, USA).

### Immunohistochemistry

An immunohistochemistry staining system from Santa Cruz (Santa Cruz Biotechnology, Inc., Santa Cruz, CA, USA) was used for detection of Fas and FasL protein in RA synovium. Grafted RA synovium samples from SCID mice were snap-frozen. The cryosections (6 μm) were fixed in cold acetone and treated with 3% hydrogen peroxide, blocked with 5% non-fat milk and 10% goat or horse serum, and incubated with the affinity-purified polyclonal rabbit anti-human Fas antibody or goat anti-FasL of mouse, rat and human antibody at room temperature for 1 h. Purified rabbit immunoglobulin G (IgG) or goat IgG was used as a control. After three washes with PBS, biotinylated goat anti-rabbit or horse anti-goat IgG was then added to the tissue. Positive staining was viewed subsequently using an avidin-peroxidase and diaminobenzidine color detection system combined with a hematoxylin counter-stain.

### Terminal deoxyribonucleotidyl transferase-mediated dUTP nick end labeling

An ApopTag fluorescein *in situ *apoptosis detection kit (Intergen Company, Purchase, NY, USA) was used to detect apoptotic cells in RA synovium. The formalin-fixed and paraffin-embedded tissue sections (6 μm) were permeabilized with proteinase K (20 μg/ml) and then the 3'-OH ends of fragmented DNA were labeled with terminal deoxyribonucleotidyl transferase. The incorporated digitonigen-dUTP was detected by incubation with fluorescein-conjugated anti-digitonigen antibody at room temperature for 60 minutes, and positive reactions were revealed under fluorescence microscopy using appropriate excitation and emission filters after propidium iodide counter-staining and mounting (Vector Laboratories Inc., Burlingame, CA, USA). The apoptotic cells and total synovial cells were counted blindly to the specific treatments at 10 high power microscopic fields randomly selected for each section. Three sections at multiple layers were examined for each sample. The percentage of apoptotic cells was recorded and represented as mean and standard deviation for each group.

## Results

### Adenovirus vector mediated dose dependent transgene expression in human synovial tissues *in vivo *in SCID mice

**Figure 1:**
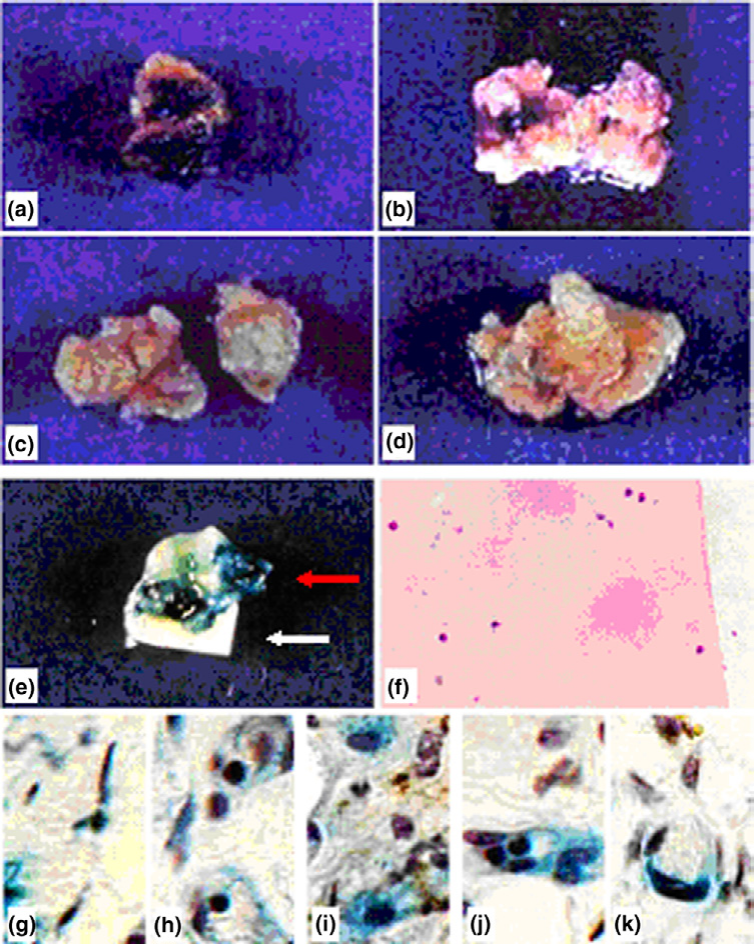
Adenovirus vector mediated LacZ transgene expression in synovial tissues. Synovium alone or synovium with cartilage from joint replacement surgery and synovectomy was implanted subcutaneously on the lower backs of C.B-17 severe combined immunodeficiency (SCID) mice. LacZ adenovirus (Ad-LacZ) in 0.1 ml PBS was injected at a dosage of 10^11^, 10^10^, or 10^9 ^particles into the middle of the grafted tissues. An equal amount of PBS was injected as a control. Three days after virus injection, synovial tissues were collected and LacZ transgene expression in the synovial tissues was detected by X-gal staining. **(a) **The dose of 10^11 ^virus particles of Ad-LacZ per injection resulted in high transgene expression in human synovium; **(b) **the dose of 10^10 ^virus particles of Ad-LacZ per injection resulted in lower transgene expression; **(c) **the dose of 10^9 ^virus particles of Ad-LacZ per injection failed to induce LacZ transgene expression, similar to **(d) **PBS injected control tissues. **(e) **The expression of LacZ transgene only was apparent in synovium but not in cartilage three days after injection of 10^11 ^virus particles (the red arrow indicates synovium; the white arrow indicates cartilage). **(f) **There was no evidence of chondrocyte infection by Ad-LacZ in cartilage, as demonstrated by X-gal/hematoxylin and eosin staining (original magnification 200×). A variety of cell types including **(g) **synoviocytes, **(h) **lymphocytes, **(i) **macrophages, **(j) **granulocytes and **(k) **endothelial cells could be infected by adenovirus vector, indicated by LacZ transgene expression detected with X-gal staining **(g-k) **(original magnification 400×).

We tested Ad-LacZ transgene expression at the dosage of 10^9^, 10^10^, or 10^11 ^particles per injection to determine the suitable experimental dosage of Ad-FasL in the *in vivo *RA-SCID model. The implanted human synovium only or with cartilage was harvested three days after virus injection and examined by X-gal staining (Fig. 1). The results show that the adenovirus vector mediates high dose dependent transgene expression in human synovium (Fig. 1a–d) but not in cartilage (Fig. 1e,f) *in vivo *three days after virus injection. The adenovirus vector infected synoviocytes, lymphocytes, macrophages, granulocytes and endothelial cells in human synovium, as indicated by LacZ transgene expression in these cell types (Fig. 1g–k). The most efficient dosage of adenovirus vector mediated gene transfer into human synovium *in vivo *was 10^11 ^particles of virus per injection (Fig. 1a). This virus dosage was selected for further *in vivo *experiments with Ad-FasL.

### Time dependent FasL transgene expression in engrafted human synovium *in vivo *in SCID mice

**Figure 2:**
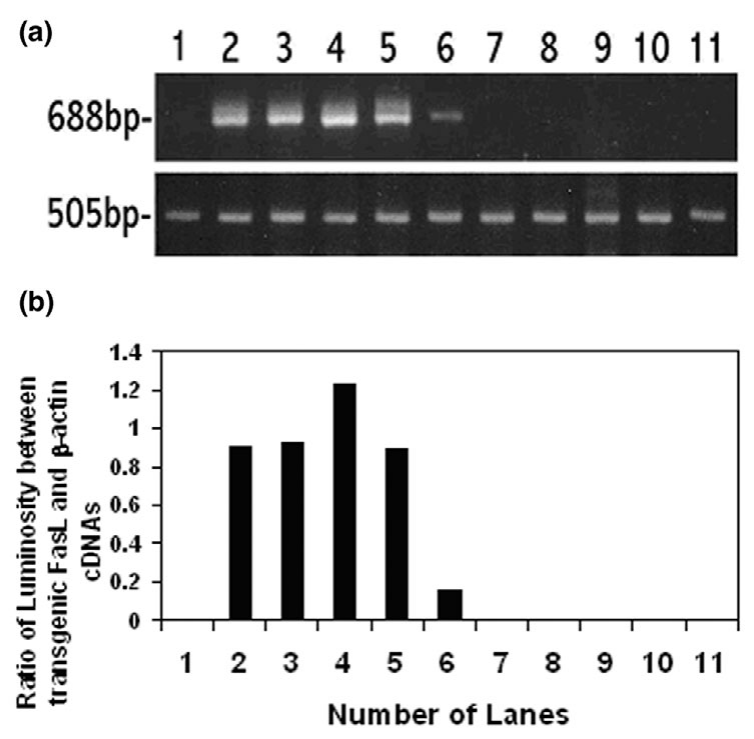
Time dependent transgenic Fas ligand (FasL) mRNA expression in human synovium *in vivo*. The 10^11 ^virus particles of recombinant FasL adenovirus (Ad-FasL) and LacZ adenovirus (Ad-LacZ) were injected into the grafted human synovium in severe combined immunodeficiency (SCID) mice *in vivo*. The injected tissue samples were harvested at days 2, 5, 7, 10, and 20 after virus injection. Total tissue RNA was isolated and the first-strand cDNA of transgenic FasL was detected by RT-PCR as described in Materials and methods. **(a) **Lane 1, PBS injected tissue; lanes 2–6, Ad-FasL infected tissues harvested on day 2, 5, 7, 10, and 20, respectively, after virus injection; lanes 7–11, the Ad-LacZ infected tissues harvested at the same time points. Ethidium bromide staining of fractionated DNA at 688 base pairs (bp) represents transgenic FasL mRNA expression; the DNA fragments at 505 bp represent β-actin expression as the internal control for the quality of mRNA samples. **(b) **Transgenic FasL mRNA *in vivo *was highly expressed on days 2–10 (lanes 2–5), and obviously decreased on day 20 (lane 6), indicated by the ratio of luminosity between transgenic FasL cDNA and the endogenous β-actin cDNA.

To investigate the adenovirus vector mediated, time dependent FasL transgene expression in engrafted human synovium *in vivo*, 10^11 ^virus particles of Ad-FasL per dose were injected into the middle of the implanted human synovium in SCID mice. The same dosage of Ad-LacZ was injected into the synovium *in vivo *as a control. Tissue samples were harvested on days 2, 5, 7, 10 and 20. Transgenic FasL mRNA expression in tissue samples was detected by RT-PCR (Fig. 2). No transgenic FasL mRNA was detected in Ad-LacZ injected tissues samples (Fig. 2, lanes 7–11). The transgenic FasL mRNA was highly expressed on days 2 to 10 and obviously decreased on day 20 after virus injection (Fig. 2, lanes 2–6), which suggests that repeated administration of FasL gene transfer would be necessary to maintain a high level of FasL transgene expression *in vivo*. This experiment was repeated and the same gene expression profile was observed. Therefore, we decided to perform repeated Ad-FasL treatment every two weeks in our *in vivo *experiments.

### FasL gene transfer increases the expression of FasL protein and the frequency of apoptotic cells in RA synovium

**Figure 3:**
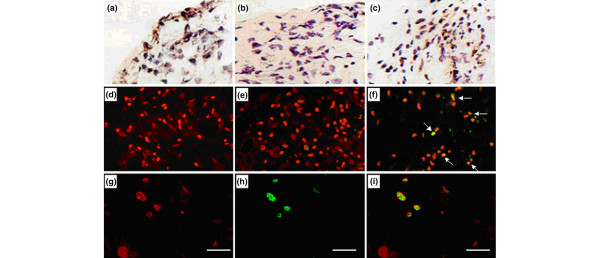
Detection of apoptotic cells and Fas/Fas ligand (FasL) protein expression in grafted rheumatoid arthritis (RA) synovium. The RA synovium in severe combined immunodeficiency (SCID) mice was harvested approximately two weeks after engraftment without or with three day treatment with 10^11 ^particles of recombinant FasL adenovirus (Ad-FasL), LacZ adenovirus (Ad-LacZ), or PBS before it was collected. **(a-c) **Fas and FasL protein expression in RA synovium *in vivo *were examined using an immunohistochemistry staining system. **(a) **Fas protein is highly expressed on synoviocytes in RA synovium (original magnification 400×). **(b) **FasL protein is lacking in RA synoviocytes treated with Ad-LacZ (original magnification 400×); **(c) **but a significant increase in FasL protein expression in RA synoviocytes appears three days after Ad-FasL injection (original magnification 400×). **(d-i) **Apoptotic cells in RA synovium were examined using the ApopTag fluorescein *in situ *apoptosis detection kit (Intergen). The red propidium iodide counter-staining indicates the nucleus of all synovial cells in RA synovium. The green fluorescein stains the fragmented DNA in the nucleus. **(d) **Apoptotic cells in PBS treated RA synovium are almost undetectable (original magnification 200×). **(e) **A slightly increased apoptotic cell population was seen in Ad-LacZ treated RA synovium (original magnification 200×), **(f) **while around a 15-fold increased frequency of apoptotic cells in RA synovium was observed in Ad-FasL treated RA synovium (original magnification 200×; arrows point to TUNEL-positive cells). **(g-i) **The location of the fragmented DNA was observed using a laser scanning confocal microscope. **(g) **The nucleus of synovial cells in RA synovium was stained with propidium iodide (red; bar = 15 μm). **(h) **Fragmented DNA was stained with fluorescein (green; bar = 15 μm). (i) The localization of the fragmented DNA was determined by the overlap of the fluorescein staining and propidium iodide counter-staining (bar = 15 μm), which characterizes cell death.

**Figure 4:**
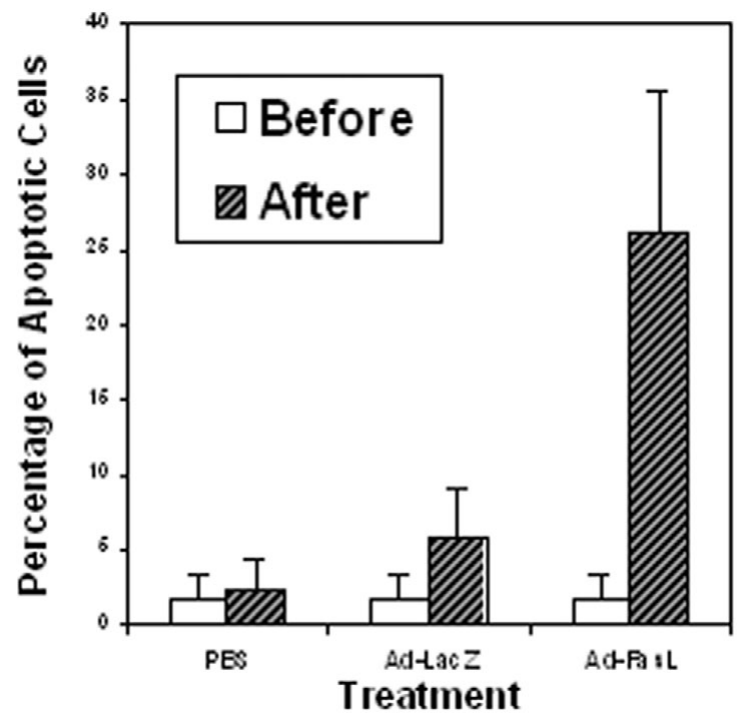
Efficient increase of apoptosis incidence in rheumatoid arthritis (RA) synoviocytes by administration of recombinant FasL adenovirus (Ad-FasL). The percentage of apoptotic cells in grafted RA synovium in severe combined immunodeficiency (SCID) mice is compared to that in the RA synovium three days after treatments with Ad-FasL, LacZ adenovirus (Ad-LacZ) and PBS. Total synoviocytes and apoptotic cells were counted in 10 high power fluorescent microscopic fields randomly selected from each section, and three sections at multiple layers for each sample were examined. The percentage of apoptotic cells was recorded and is represented as the mean and standard deviation for each group. Open bars represent the percentage of apoptotic cells in RA synovium two weeks after engraftment without treatment; striped bars represent the percentage of apoptotic cells in RA synovium three days after Ad-FasL, Ad-LacZ and PBS injections.

Fas protein is highly expressed in RA synovial lining cells and almost all kinds of inflammatory cells infiltrating the synovium, but FasL protein is poorly expressed in RA synovium [11]. We determined Fas and FasL protein expression in engrafted RA synovium by immunohistochemistry using polyclonal anti-Fas and anti-FasL antibodies. Our experimental results (Fig. 3a,b) are similar to those published by another group [11]. Three days after Ad-FasL gene transfer into RA synovium *in vivo*, augmented FasL protein expression was observed (Fig. 3c), and an increased frequency of apoptotic cells in RA synovium was detected by terminal deoxyribonucleotidyl transferase-mediated dUTP nick end labeling (TUNEL) staining (Fig. 3f). DNA fragmentation was determined in the location of the nucleus using a Leica TCS SPII laser scanning confocal microscope (Leica Microsystems, Heidelberg, Germany) (Fig. 3g–i). Few apoptotic cells were detected in RA synovium in the SCID mouse *in vivo *two weeks after tissue grafting. Three days after Ad-FasL gene transfer, the frequency of apoptotic cells increased 15 to 20-fold (Fig. 4). The frequency of apoptotic cells in PBS treated RA synovium underwent almost no change, and the frequency of apoptotic cells in Ad-LacZ injected RA synovium increased only slightly (Fig. 4).

### Elimination of inflammatory RA synovium *in vivo*

**Figure 5:**
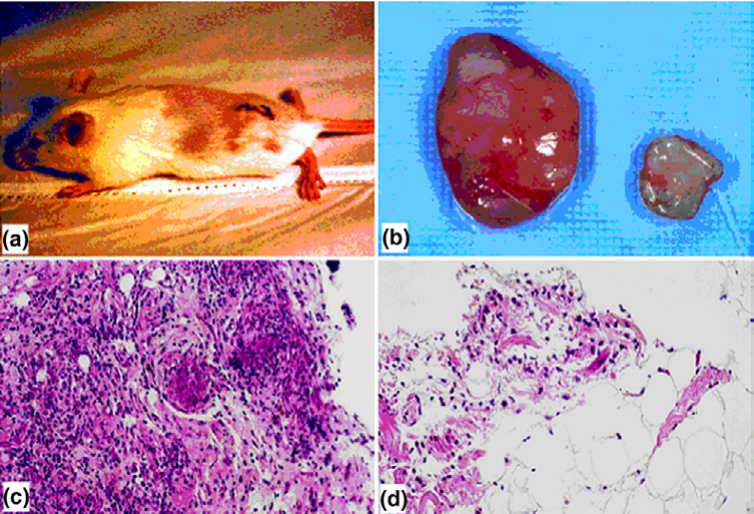
Reduction of the size and cell density of rheumatoid arthritis (RA) synovium *in vivo *by repeated Fas ligand (FasL) gene transfer. **(a) **RA synovium, 200 mg tissue per mouse, was grafted into the back of each severe combined immunodeficiency (SCID) mouse subcutaneously. Two weeks after engraftment, 10^11 ^particles of recombinant FasL adenovirus (Ad-FasL) or Ad-LacZ adenovirus (Ad-LacZ) in 0.1 ml PBS were injected into the engrafted RA synovium in the SCID mice. Injections were repeated every two weeks for a total of five times. Three days after the fifth injection, the mice were euthanized and the RA synovium samples were collected, weighed, and examined with hematoxylin and eosin (H&E) staining. **(b) **A comparative view of the size of the grafted RA synovium after two months of repeated treatment with control Ad-LacZ (tissue on the left side) and Ad-FasL (tissue on the right side). **(c) **H&E staining shows that both synoviocytes and mononuclear cells are greatly decreased in Ad-FasL treated RA synovium, in which a partial recovery of the normal features of synovium was observed (original magnification 200×); **(d) **but the Ad-LacZ treated RA synovium maintains the inflammatory infiltrates (original magnification 200×)

Upregulation of Fas/FasL interaction in arthritic joints by virus vector mediated FasL gene transfer or anti-Fas antibody treatment intra-articularly can induce apoptosis in inflammatory synoviocytes, as has been identified by many groups [5,12-18]. Here the effect of continuous activation of Fas/FasL interaction through a repeated FasL gene transfer was explored to determine if this treatment could result in the death of inflammatory synoviocytes in RA synovium and elimination of RA tissue *in vivo*. The *in vivo *experiments were performed as explained in the Materials and methods section. Compared to the control Ad-LacZ treated RA synovium, the Ad-FasL injected RA-synovium was dramatically reduced in size (Fig. 5b) and weight (Table 1).

A significance test for the effects of Ad-LacZ and Ad-FasL treatments on the weight of RA synovium was performed using the rank sum test (n1 = n2 = 10, T1 = 154, T2 = 56, P < 0.005) (Table 1). Along with the approximate 15-fold increase in the frequency of apoptotic cells observed in the RA synovium three days after after Ad-FasL injection (Fig. 4) the number of both synoviocytes and mononuclear cells was greatly decreased in RA synovium after two months of treatment with repeated FasL gene transfers (Fig. 5d) compared with Ad-LacZ treated RA synovium (Fig. 5c). The RA synovium partially recovered, exhibiting features of normal human synovium after the two-month treatment with Ad-FasL (Fig. 5d).

## Discussion

Molecular surgery to remove the pathological cells and tissues *in situ *by gene transfer could be an important part of molecular medicine. Molecular synovectomy using gene scalpels simplifies the current problems of gene delivery, gene target and gene expression regulation involved in human gene therapy. Because the induction of apoptosis in inflammatory synoviocytes is the main goal for intra-articular gene transfer, a transient, localized, immune tolerant and dose dependent gene transfer may be achieved by direct intra-articular injection of apoptosis inducers carried by a suitable vector, controlled to infect only synovial cells but not chondrocytes in cartilage. In this study, we have established that the inflammatory synovium from RA patients can be effectively reduced *in vivo *in a RA-SCID mouse model by repeated, locally administered adenovirus vector mediated FasL gene transfer, strongly supporting the gene scalpel concept.

C.B-17 SCID mice have severe combined immunodeficiency, resulting from a mutation on chromosome 16 responsible for deficient activity of an enzyme involved in DNA repair [25]. We chose to use C.B-17 SCID mice in this study because this strain has successfully hosted human synovium and has provided an ideal *in vivo *experimental environment to test how human inflammatory synovium responds to novel therapeutic reagents [21-23]. Unlike C.B-17 SCID beige mice, C.B-17 SCID mice have no deficiency of macrophages and natural killer cells, which is important for our experimental design because macrophage phagocytosis of apoptotic cells [26-28] is considered as one of the important mechanisms involved in the molecular synovectomy procedure. In theory, inflammatory synoviocytes in implanted RA synovium in SCID mice may consist of antigen specific human lymphocytes, proliferated human fibroblast-like synoviocytes, and infiltrated mouse macrophages and so on. An artificial feature of this model, however, is the absence of circulating human blood components when studying the properties of rheumatoid synovium [21]. In our experiments, the inflammatory histological features of rheumatoid synovium are preserved in implanted RA synovium in SCID mice. This combination of RA synovium and SCID mice allows for repeated FasL gene transfer mediated by adenovirus vector into RA synovium *in vivo *because the C.B-17 SCID mice lack an immune response to the adenovirus vector.

The elimination of synoviocytes from RA synovium after multiple applications of FasL gene transfer results in a significant reduction in RA synovium size and weight *in vivo *in the RA-SCID mice (Fig. 5; Table 1) along with dramatic synovial cell death detected by TUNEL staining (Figs 3g–I and 4). Recombinant adenovirus is a highly efficient vector for gene transfer into the synovium [5,7,12,19]. Adenovirus vector can infect a wide variety of cell types in human synovium including synoviocytes, lymphocytes, macrophages, granulocytes and endothelial cells, as determined by X-gal staining (Fig. 1g–k). In recent studies, endothelial cells were reported to constitutively express FasL and release soluble FasL [29-31]. These cells are not sensitive to Fas-mediated apoptosis and may play a role in the negative regulation of inflammation [30]. The mediation of cell death for lymphocytes and granulocytes strongly involves the Fas/FasL apoptosis pathway [32-35].

Primary cultured macrophages can survive 1 to 2 weeks after infection with 10^4 ^particles per cell of Ad-FasL (data not shown). Fas-associated death domain-like interleukin 1beta-converting enzyme (FLICE)-inhibitory protein (Flip), a negative regulator of Fas-induced apoptosis, is upregulated when monocytes differentiate into macrophages, which may confer the resistance macrophages have to Fas-mediated apoptosis [36,37]. The survival of macrophages after FasL gene transfer may be necessary for the phagocytosis of apoptotic cells during 'molecular synovectomy' using the 'FasL gene scalpel'. On the other hand, the survival of macrophages carrying viral vector antigens may activate the naive lymphocytes and initiate an anti-virus immune response if the adenovirus vector was used for clinical administration. Thus, the development of an immune tolerant gene delivery vehicle is an important task for the clinical use of FasL gene scalpel.

Compared with Ad-LacZ gene transfer, Ad-FasL gene transfer induces a significantly increased frequency of apoptotic cells in RA synovium (Figs 3e,f and 4), even though adenovirus vector itself shows a slight effect on enhancing apoptosis in synoviocytes (Fig. 4) and certain other kinds of cells [38,39]. From the point of view that repeated FasL gene transfer can remove RA synovium *in vivo *(Fig. 5b; Table 1), the FasL gene may function as a sharp scalpel for molecular synovectomy. It has been reported that Fas-mediated apoptosis is associated with activation of the Fas-associated death domain protein/Caspase-8/Caspase-3/poly(ADP-ribose)polymerase pathway and the c-Jun amino-terminal kinase/activator protein-1 pathway [40,41]. The former is thought to be the major signaling pathway required for Fas-mediated apoptosis in RA synoviocytes; the latter appears to be involved in the pathogeneses of inflammation by inducing pro-inflammatory cytokine/chemokine production [40,42]. Thus, the clinical potential of a FasL gene scalpel will depend on the clarification of the side effects of FasL intra-articular gene transfer. The combined administration of the intra-articular gene transfer of FasL with other genes and/or certain anti-inflammatory drugs might be an ideal complementary therapeutic approach for the local treatment of RA and other arthropathies.

A subset of chondrocytes located in the superficial zone of cartilage expresses the Fas antigen, and activation of the Fas receptor triggers apoptosis in these cells [43,44]. An adenovirus mediated short-term (24 h) FasL gene transfer *in vivo *in the rabbit knee only transduced synovium, but it did not affect the viability of chondrocytes in cartilage [12]. Our *in vivo *experiments with Ad-LacZ gene transfer on human synovium and cartilage in SCID mice presents similar results (Fig. 1e,f), but after a long-term (4 weeks) *in vitro *culture the chondrocytes in the superficial zone of cartilage were infected by Ad-LacZ 24 h after virus infection (unpublished data). This phenomenon suggests that the loss of cartilage matrix may have occurred during long-term *in vitro *culture. The cartilage matrix may be important for protection of chondrocytes from infection by adenovirus vector mediated gene transfer. Thus, the most suitable period for the treatment of rheumatoid arthritis with gene transfer would be in the early stage of the disease when the cartilage matrix is undamaged. The effects of long-term gene transfer of an apoptosis inducer such as FasL on the chondrocytes in cartilage *in vivo *require further investigation.

Currently, potential strategies for the treatment of RA using intra-articular gene transfer are focused on reducing the production and activation of inflammatory cytokines/chemokines and proteases/adhesion molecules, and inducing apoptosis of inflammatory synoviocytes. The latter may be more efficient in suppressing the inflammation by removing the producing and reactive cells responding to pro-inflammatory molecules. This potential therapeutic approach using 'gene scalpels' may function as a molecular synovectomy, not only for the treatment of RA, but also for the treatment of other arthropathies.

## Conclusion

Our study elucidates firstly the *in vivo *therapeutic potential of molecular synovectomy using 'gene scalpels' for the treatment of RA locally through repeated intra-articular gene transfer of an apoptosis inducer. This approach may more efficiently arrest intra-articular inflammation and suppress the progress of RA by removing the cells producing and/or responding to pro-inflammatory molecules in arthritic joints.

## Abbreviations

Ad-FasL = recombinant FasL adenovirus; Ad-LacZ = LacZ adenovirus; bp = base pair; FasL = Fas ligand; PBS = phosphate buffered saline; RA = rheumatoid arthritis; RT-PCR = reverse transcription-polymerase chain reaction; SCID = severe combined immunodeficiency; TUNEL = terminal deoxyribonucleotidyl transferase-mediated dUTP nick end labeling; X-gal = 5-bromo-4-chloro-3-indolyl β-d-galactopyranoside.

## Competing interests

The author(s) declare that they have no competing interests.

## Authors' contributions

GG provided the adenoviruses for study. GC contributed to the experiments and generated the histology data. RS coordinated the collection of the synovial tissues from patients in the clinic, carried out the pathological diagnosis of the human samples and provided advisory support for the study. HZ conceived of the study, participated in its design and coordination, carry out the *in vivo *study and molecular examinations, and drafted the manuscript. All authors read and approved the final manuscript.

## Figures and Tables

**Table 1:** Effect of repeated Ad-FasL or Ad-LacZ treatment on the weight of RA synovium

Ad-LacZ treated		Ad-FasL treated	
Weight (mg)	Rank no.	Weight (mg)	Rank no.

162.00	16	17.00	4
122.00	13	72.00	11
171.00	17	29.00	6
159.00	15	62.00	9
203.00	20	11.00	3
184.00	19	46.00	8
98.00	12	34.00	7
177.00	18	10.00	2
156.00	14	0.00	1
68.00	10	25.00	5
n1 = 10	T1 = 154	n2 = 10	T2 = 56
P < 0.005 (Rank sum test^a^)			

The repeated gene transfer of recombinant FasL adenovirus (Ad-FasL) and LacZ adenovirus (Ad-LacZ) into rheumatoid arthritis (RA) synovium in severe combined immunodeficiency (SCID) mice are the same as described in Fig. 5. Ten samples per treatment group were examined. After two months of treatment with viruses, the RA synovium samples were collected. The weight of each sample was measured individually for each group. ^a^The rank sum test was used to determine the P-value (P < 0.005).
